# Diel nitrogen fixation pattern of *Trichodesmium*: the interactive control of light and Ni

**DOI:** 10.1038/srep04445

**Published:** 2014-03-24

**Authors:** Irene B. Rodriguez, Tung-Yuan Ho

**Affiliations:** 1 Research Center for Environmental Changes, Academia Sinica, Taipei, Taiwan

## Abstract

*Trichodesmium*, a nonheterocystous cyanobacterium widely abundant in the surface water of the tropical and subtropical ocean, fixes dinitrogen under high light conditions while concurrently undergoing photosynthesis. The new production considerably influences the cycling of nitrogen and carbon in the ocean. Here, we investigated how light intensity and nickel (Ni) availability interplay to control daily rates and diel patterns of N_2_ fixation in *Trichodesmium*. We found that increasing Ni concentration increased N_2_ fixation rates by up to 30-fold in the high light treatment. Cultures subjected to high Ni and light levels fixed nitrogen throughout most of the 24 H light:dark regime with the highest rate coinciding with the end of the 12 H light period. Our study demonstrates the importance of Ni on nitrogen fixation rates for *Trichodesmium* under high light conditions.

Nitrogen fixation is a critical process to provide new bioavailable nitrogen to phytoplankton in the ocean^1^,^2^,^3^,^4^,^5^, where nitrogen is considered to be the most important limiting nutrient for phytoplankton growth in the ocean. *Trichodesmium*, a filamentous cyanobacterium, is a particularly important oceanic diazotroph due to its substantial contribution of fixed nitrogen and new production to the tropical and subtropical ocean^1^,^2^. Previous laboratory and field studies indicate that the nitrogen fixing ability of *Trichodesmium* may be controlled by the supply of Fe and P in the surface waters^6^,^7^,^8^. Our recent laboratory studies show that Ni availability also exhibits a crucial role in the nitrogen fixing process of *Trichodesmium*, most likely due to the presence of Ni in Ni-superoxide dismutase, which is needed to protect nitrogenase from reactive oxygen species (ROS) generated through photosynthesis^9^,^10^. These two studies indicate that the sufficient supply of Ni is essential to sustain the growth of *Trichodesmium*^9^, particularly under high light conditions^10^. The light intensities in the surface water of the tropical and subtropical ocean, which *Trichodesmium* inhabits, can reach ~2000 μE m^−2^ s^−1^ during high noon in summer. In addition, both laboratory and field studies have shown that the growth and nitrogen fixation rates of *Trichodesmium* are positively correlated with light intensity^11^,^12^. *Trichodesmium*, a nonheterocystous cyanobacterium, carries out oxygen-producing photosynthesis and nitrogen fixation simultaneously during the light period. This photosynthetic O_2_ production is problematic as nitrogenase is known to be irreversibly inactivated by oxygen and other ROS^13^,^14^. The mechanisms that *Trichodesmium* possesses to simultaneously carry out nitrogen fixation and photosynthesis in a strong light environment remain unclear.

## Results

**Figure 1:**
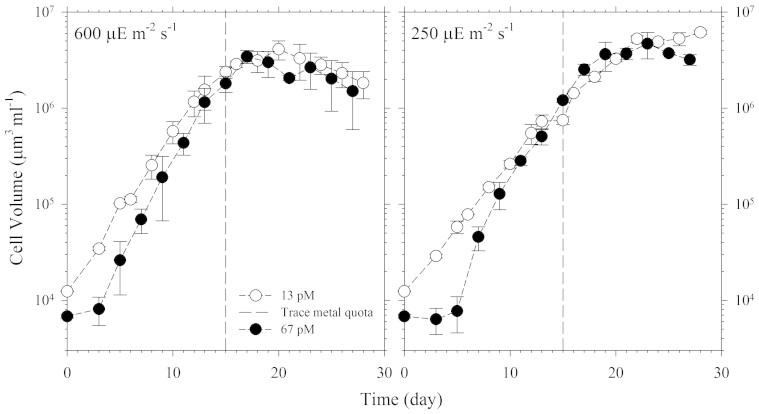
Growth curves and cellular biomass of the *Trichodesmium* cultures subjected to the light intensities at 600 and 250 μE m^−2^ s^−1^. The cultures were grown in trace-metal defined medium at inorganic Ni concentrations to be 67 and 13 pM Ni′ (solid and open circles). Nitrogen fixation experiments were conducted while the cells were at the exponential phase. Error bars represent standard deviation of three replicate culture bottles. The dashed line indicates the date when culture samples for intracellular metal quotas were collected.

**Figure 2:**
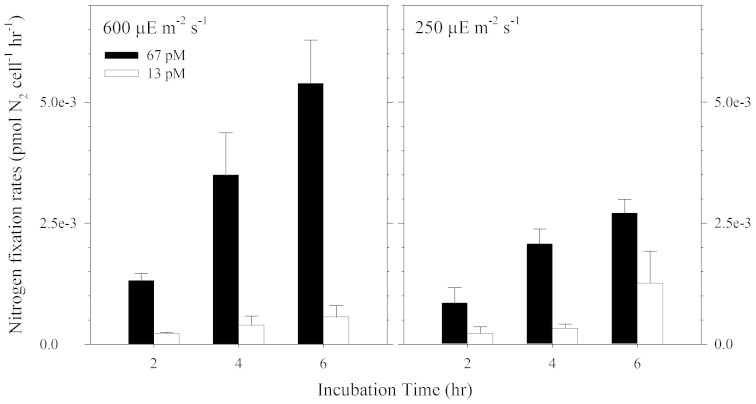
Nitrogen fixation rates by *Trichodesmium* cultures subjected to the light intensities at 600 and 250 μE m^−2^ s^−1^. The cultures were grown in trace-metal defined medium at Ni′ to be 67 and 13 pM (solid and open bars). The cultures were kept at 12:12H light:dark cycle in a temperature-controlled growth chamber maintained at 26°C. Error bars represent standard deviation of triplicate culture bottles.

We carried out two culture experiments to investigate the coupled effect of light intensity and Ni availability on nitrogen fixation rates and cellular growth rates (see details in Methods section). The effect of light intensity and the concentration of biologically available dissolved inorganic Ni species (referred to here as Ni′) on nitrogen fixation rates were determined over a 6H light period and a 24H light/dark cycle. We observed that the cellular growth rate in the high light and Ni-sufficient treatment was slightly higher than rates observed in the lower light treatments (Fig. 1). The growth rates obtained in cultures subjected to 250 μE m^−2^ s^−1^ were 0.33 and 0.35 day^−1^ for the treatments with 13 and 67 pM Ni′, respectively. The growth rates in cultures subjected to a light intensity of 600 μE m^−2^ s^−1^ were higher, 0.40 and 0.48 day^−1^ respectively, for the low and high-Ni treatments. These growth rates were determined prior to collection of cells for trace metal quota determination and while the cells were in the exponential growth phase. The influence of light intensity on growth rates was consistent with previous laboratory and field observations that high light enhances *Trichodesmium* growth^10^,^11^,^12^. The 6H acetylene reduction assay shows that at the lower light intensity, 250 μE m^−2^ s^−1^, the nitrogen fixation rates were 2 to 6 times higher in cultures with relatively high Ni availability (Ni′ = 67 pM) than the cultures with relatively low Ni availability (Ni′ = 13 pM) (Fig. 2). At the relatively high light intensity, 600 μE m^−2^ s^−1^, the nitrogen fixation rates for the high Ni cultures were 6 to 10 times higher than in the low Ni cultures (Fig. 2). The observed increase in nitrogen fixation rates by *Trichodesmium* with increasing Ni concentration indicates that the capacity to fix nitrogen is limited by Ni availability, especially at high light intensities.

**Figure 3:**
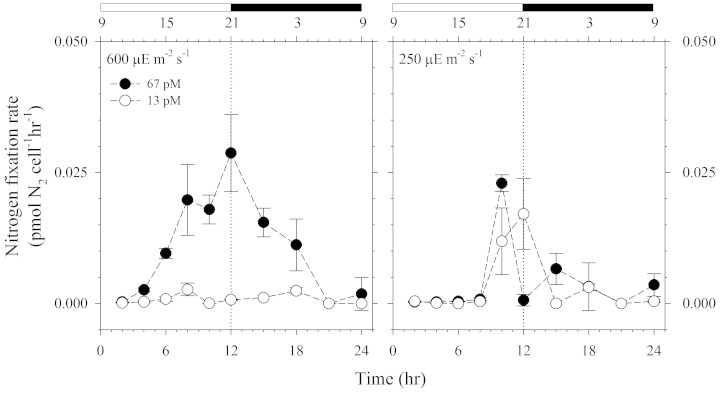
Diel nitrogen fixation rates by *Trichodesmium* cultures subjected to the light intensities at 600 and 250 μE m^−2^ s^−1^. The cultures were kept at 12:12H light:dark cycle represented by light and dark bars on top of the graphs. Error bars represent the standard deviation of three replicate culture bottles.

The first experiment covered only 6H during the middle of the photoperiod and was initiated 2 hours after the light period commenced. We then carried out a second experiment employing similar conditions as the 6H experiment to determine nitrogen fixation rates for an entire 24H light:dark diurnal cycle. In agreement with the results observed from the 6H experiment, we found that the Ni sufficient cultures (Ni′ = 67 pM) achieved much higher rates, as much as 30 times higher, than the low-Ni cultures (Ni′ = 13 pM) at the high light intensity (Fig. 3). The effect of Ni was less striking at the relatively low light intensity used (Fig. 3). The results of the 24H experiment further validate the hypothesis that Ni availability and light intensity interact to influence the nitrogen fixation in *Trichodesmium*^10^. More importantly, the 24H experiment revealed a new diel pattern of nitrogen fixation, exhibiting the maximum rate near or at the end of the light phase and with the nitrogen fixation process sustained during the dark phase for the high Ni-high light treatment (Fig. 3). This unique temporal profile is in contrast to what was observed for cultures at the lower light intensity (250 μE m^−2^ s^−1^), where the window of nitrogen fixation only occurred for a short period near or at the end of the light phase.

## Discussion

The diel nitrogen fixation profiles we have observed in this study differ from the pattern reported in previous works, where the nitrogen fixing activities were confined to day time and the maximum rates occurred at the middle of the photoperiod^15^,^16^,^17^,^18^. It should be noted that most of the previous culture studies were carried out at lower light intensities ranging from 30 to 200 μE m^−2^ s^−1^, and that Ni availability was not specifically controlled in their culture media^15^,^16^,^17^,^18^. Indeed, Ni is not included in the nutrient recipe of YBC II, the major culture medium used for *Trichodesmium*^19^. Ho (2013) shows that the YBC II medium could still contain relatively high Ni even without intentional Ni addition^9^. Our results highlight the need for *Trichodesmium* to have mechanisms to protect nitrogenase, the key enzyme in nitrogen fixation that is susceptible to damage from photosynthetically released O_2_ and ROS, because the energy-demanding diazotrophic process is carried out during the day time^1^. A previous study suggested that *Trichodesmium* may down regulate photosynthesis during high light at noon time to permit high nitrogenase activity^15^, which may allow the diazotroph to fix nitrogen under lower rates of oxygen production. However, the temporal down regulating strategy does not explain why the growth rates and nitrogen fixation rates increase with light intensity in the present and past experiments^9^,^10^,^11^,^12^. Here, we found that the nitrogen fixing process was not confined to the photoperiod when *Trichodesmium* was subjected to intense 12H light exposure and sufficient Ni supply. Our study indicates that *Trichodesmium* possesses a mechanism in place to allow it to concomitantly fix carbon and nitrogen under high light conditions then derive its energy requirement to support nitrogen fixation in the dark. During the light phase, *Trichodesmium* may additionally take advantage of the presence of Ni-Fe uptake hydrogenase^20^,^21^. The Ni-Fe uptake hydrogenase increases the efficiency of N_2_ fixation by catalyzing H_2_, which produces both ATP and reducing equivalents that can be used to fix more N_2_. In the dark, the most likely primary source of energy needed for N_2_ fixation is respiration of organic carbon produced by photosynthesis. Both the hydrogenase reaction and the respiration of organic matter consume large amounts of O_2_, which also helps prevent O_2_ poisoning of nitrogenase. The existence of the genes encoding Ni-SOD^22^ and Ni-Fe uptake hydrogenases implies that *Trichodesmium* might be utilizing both to promote optimal rates of N_2_ fixation during both day and night.

**Figure 4:**
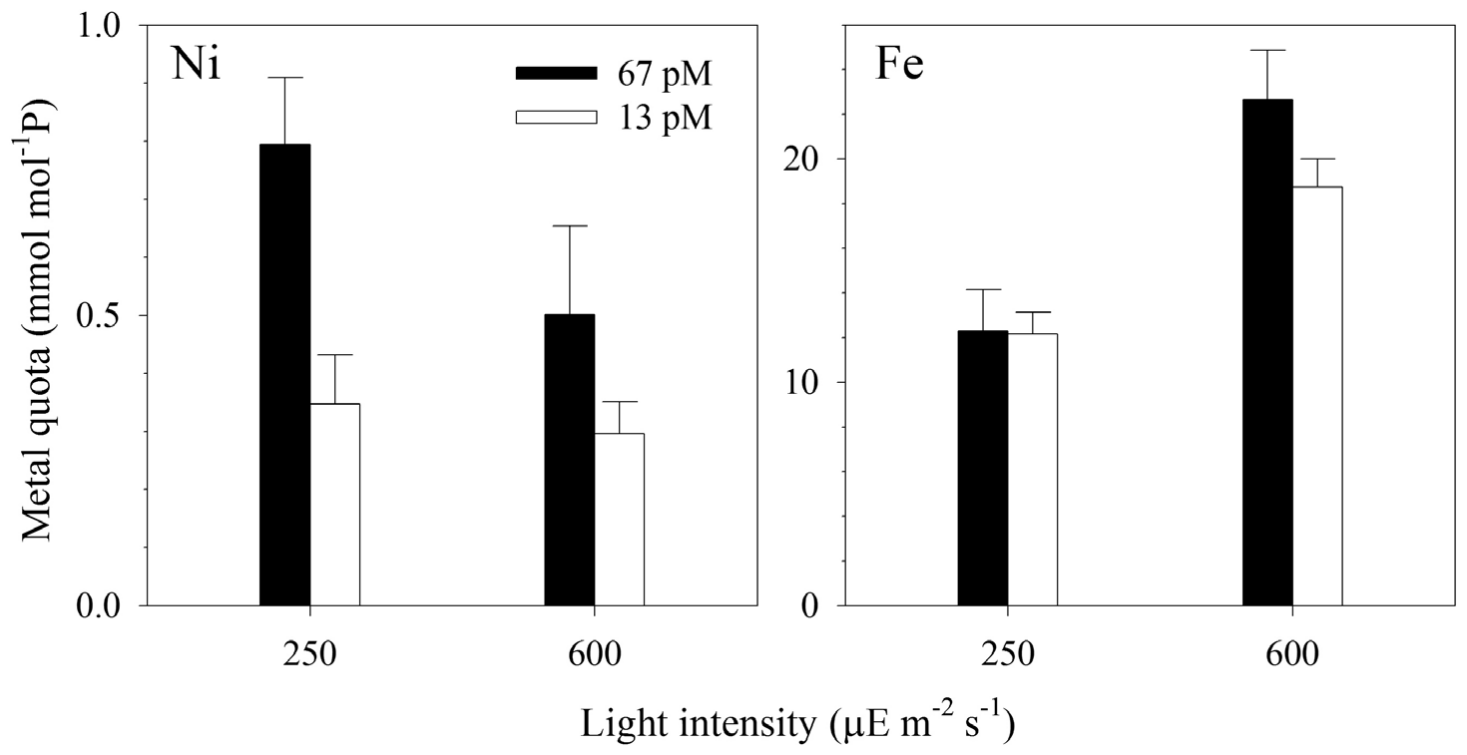
Intracellular trace metal quota of *Trichodesmium* cultures subjected to the light intensities at 600 and 250 μE m^−2^ s^−1^. Trace metal quota were normalized to the phosphorus content of the cells (mmol/mol P). Although we targeted to harvest the cells at the exponential phase, the culture samples from the high light treatment were already close to the stationary phase (Fig. 1). Error bars represent the standard deviation of three replicate culture bottles.

The cellular metal quotas of Fe and Ni were shown by normalizing their total intracellular concentrations to phosphorus as a biomass proxy (Fig. 4). The cellular Fe:P ratios increased by a factor of 1.5 with the increase in light intensity. These cellular iron quotas may be associated with the biochemical iron requirements for nitrogen fixation through the associated Fe containing enzymes, such as nitrogenase^23^ and various Fe enzymes of the photosynthetic apparatus^24^. In addition, the higher Fe:P ratio is also likely attributed to the higher iron availability to the *Trichodesmium* under higher light intensity, which is linked to photo redox cycling of Fe(III)-EDTA chelates^25^,^26^,^27^. Because the cycling rates are proportional to light intensity, the higher light intensity would result in higher cellular iron uptake rates by increasing steady-state concentrations of dissolved inorganic ferrous and ferric iron species^25^,^26^,^27^. The cell Ni:P ratios in the high-Ni cultures were about 2-fold higher than those in the low-Ni cultures. However, the Ni quotas of the cultures at high light intensity (600 μE m^−2^ s^−1^) were comparable or slightly lower than those in the low light cultures. We had expected that the increase in light intensities should lead to elevated levels of ROS, which should have entailed the use of even more Ni-SOD and elevated uptake for Ni. The cellular metal concentration is equal to the uptake rate divided by the specific growth rate, thus when the growth rates decline, the cellular metal levels will increase provided that the metal uptake rate remains constant^28^. We found that the specific growth rates of the high Ni treatments were 0.40 and 0.48 d^−1^ for the growth period prior to the measurement of Ni quota under the low and high light conditions, respectively. If the Ni uptake rate remained constant, that difference in the growth rates would result in a 20% increase in the cellular Ni quota, explaining most of the observed increase in the quota.

Our study demonstrates the importance of Ni on nitrogen fixation rates for *Trichodesmium* under high light conditions. A 12-fold increase was observed for cultures with the high light-high Ni treatment compared to the cultures with low Ni treatment for the 24H period. Aside from the marked increase in total nitrogen fixed, the increase in both Ni and light may extend the nitrogen fixation process beyond the photoperiod. The effect of Ni is related to the role of Ni-SOD in reducing oxidative stress by removing superoxide radicals, and in Ni-Fe uptake hydrogenases, which increases the efficiency of N_2_ fixation by utilizing the H_2_ released as by-product of N_2_ fixation. The extent to which Ni regulates N_2_- and C-fixation in *Trichodesmium* in the ocean needs to be investigated to further understand how Ni influences the occurrence and distribution of this diazotroph in the ocean. Further field enrichment experiments should be carried out to test whether Ni may be a limiting factor in the tropical and subtropical oceans, particularly under high light conditions. It would also be interesting to learn whether the 2 nM dissolved Ni generally observed in the surface water of the subtropical and tropical oceans are bioavailable to *Trichodesmium*, and whether the nitrogen fixation rates and the abundance of *Trichodesmium* are regulated by bioavailable Ni concentrations. The findings of these studies may be important for understanding the distributions and activities of *Trichodesmium* and the environmental controls on its nitrogen fixation in modern and ancient oceans.

## Methods

### *Trichodesmium* cultures

Materials used for culturing were carefully washed with Micro-90® solution, rinsed, soaked with 10% hydrochloric acid solution, and rinsed thoroughly with superpure Milli-Q water. All necessary procedures including the medium preparation, culturing, and harvesting of cells for trace metal quota determination were carried out in a class 100 trace-metal clean laboratory. The nonaxenic cultures of *Trichodesmium erythraeum* (obtained from the National Center for Marine Alga and Microbiota) were grown in 1 L trace metal clean polycarbonate bottles (Nalgene, USA) with a trace metal-defined medium^9^ modified from the original recipe^19^. The medium was passed through Chelex-100 resin prior to the addition of trace metals. The dissolved total concentrations of the trace metals were at the following values: Fe, Mo, Mn, Zn, Co, Se, and Cu at 400, 100, 10, 10, 10, and 10 nM, respectively. The availability of the trace metals added was controlled by adding 20 μM of ethylenediaminetetraacetic acid (EDTA). The two different Ni treatments were achieved by adding total Ni concentrations of 20 and 100 nM, which resulted to 13 pM and 67 pM respectively of inorganic Ni (Ni′) in the culture media^9^,^10^. The total initial concentration of P was 50 μM and the B-vitamins were added at the suggested levels^19^. These culture conditions were designed such that P and Fe levels are sufficient in the culture medium. The cultures were kept in a temperature-controlled growth chamber fixed at 26°C with different light treatments at 250 and 600 μE m^−2^ s^−1^. Photon irradiances were achieved by placing the culture bottles in appropriate distances from the light source, and were verified by measuring the light penetration PAR into a seawater-filled polycarbonate bottle using a submersible radiometer (Biospherical Instruments Inc. QSL 2100). The growth chamber was kept at a 12:12 H light:dark cycle. All sets of treatments were carried out in triplicates.

### Growth rates and intracellular metal quota

The growth of the cultures was monitored by measuring the total cellular volume using a Beckman Coulter Counter Multisizer 3 until decline in the biomass was observed for all culture bottles^9^,^29^. As explained elsewhere^9^, the use of the Coulter counter provides a precise and reliable way of monitoring growth rates. The growth rates were determined on different days, between days 5 to 13, during the log-linear stage of the growth curve. Determination of intracellular quotas was done by harvesting *Trichodesmium* cells while on the exponential phase of the growth curve (indicated in Fig. 1). The cells were filtered onto acid washed polycarbonate filters (25 mm with 5 μm pore size), washed with ultrapure Milli-Q water and subsequently decomposed before analysis^9^,^10^. The elemental composition was determined using HR-ICPMS (Element XR, Thermo Scientific).

### Nitrogen fixation rates

The nitrogen fixation rates were estimated using the acetylene reduction method^30^ following the steps outlined elsewhere^9^,^30^. Two sets of experiments were conducted to elucidate the effect of Ni availability and light intensity on the N_2_ fixation rates. The first experiment was for a short incubation period lasting for 6H and was intended to study the rates while the second was designed to cover a 24H period to study the diel pattern of N_2_ fixation. In brief, 10 ml aliquots of the cultures (duplicate samples were prepared from each of the triplicate bottles per treatment for each time point) were transferred to 20 ml vials (Agilent). The vials were sealed using Teflon-coated caps and 2 ml air was drawn using a syringe. The air in the sealed vials was replaced by 2 ml of freshly-prepared acetylene to initiate the experiment. The vials were then incubated at the same growth conditions for 2 to 6 hours in the 6H experiment and 2 to 24 hours for the 24H experiment. The time-point experiment was designed so that nitrogen fixation was stopped after every 2 hours during the light phase (for both experiments) and after every 3 hours during the dark phase (for the 24H experiment). The nitrogen fixation experiment was started to coincide with the start of the light phase, which commenced at 9AM, of the light:dark cycle. After incubation for the desired period, 2 ml of headspace was drawn and the gaseous sample was subsequently analyzed for ethylene using an Agilent 7890A gas chromatograph equipped with a Poropak N column (Agilent) and a flame ionization detector. Estimation of the dinitrogen reduction was taken from the acetylene reduction using a conversion ratio of 4:1, and the assumption that the Bunsen coefficient for ethylene is 0.084^31^. Nitrogen fixation experiments were conducted while cells were at the exponential growth stage. The rate at a specific time point was calculated by subtracting the accumulated C_2_H_4_ at the preceding point divided by the duration.

## Author Contributions

T.Y.H. conceptualized the study. I.B.R. and T.Y.H. planned and designed the study; I.B.R. carried out the experiments; I.B.R. and T.Y.H. analyzed the data and wrote the paper.
